# Seasonal spatial-temporal trends of vegetation recovery in burned areas across Africa

**DOI:** 10.1371/journal.pone.0316472

**Published:** 2025-02-03

**Authors:** Oswaldo Maillard, Natasha Ribeiro, Amanda Armstrong, Ana I. Ribeiro-Barros, Samora Macrice Andrew, Lucy Amissah, Zeinab Shirvani, Jonathan Muledi, Omid Abdi, Huascar Azurduy, João M. N. Silva, Stephen Syampungani, Hastings Shamaoma, Victorino Buramuge

**Affiliations:** 1 Fundación para la Conservación del Bosque Chiquitano, Santa Cruz, Bolivia; 2 College of Agriculture and Forestry, University of Eduardo Mondlane, Maputo, Mozambique; 3 Earth System Science Interdisciplinary Center, University of Maryland, College Park, Maryland, United States of America; 4 NASA Goddard Space Flight Center, Greenbelt, Maryland, United States of America; 5 Forest Research Centre, Associate Laboratory TERRA, School of Agriculture, University of Lisbon, Lisbon, Portugal; 6 College of Natural and Applied Sciences, University of Dar es Salaam, Dar es Salaam, Tanzania; 7 CSIR-Forestry Research Institute of Ghana, Kumasi, Ghana; 8 Division of Geoinformatics, KTH Royal Institute of Technology, Stockholm, Sweden; 9 Ecologie, Restauration Ecologique et Paysage, Faculté des sciences agronomiques et environnement, Université de Lubumbashi, Lubumbashi, République Démocratique du Congo; 10 Department of Forest Sciences, University of Helsinki, Helsinki, Finland; 11 ORTARChI Chair of Environment and Development, Copperbelt University, Kitwe, Zambia; 12 University of Pretoria, Department of Plant and Soil Sciences, Pretoria, South Africa; 13 Copperbelt University, School of Built Environment, Kitwe, Zambia; City College of New York, UNITED STATES OF AMERICA

## Abstract

Africa is entering a new fire paradigm, with climate change and increasing anthropogenic pressure shifting the patterns of frequency and severity. Thus, it is crucial to use available information and technologies to understand vegetation dynamics during the post-fire recovery processes. The main objective of this study was to evaluate the seasonal spatio-temporal trends of vegetation recovery in response to fires across Africa, from 2001 to 2020. Non-parametric tests were used to analyze MODIS Normalized Difference Vegetation Index (NDVI) products comparing the following three-month seasonal periods: December-February (DJF), March-May (MAM), June-August (JJA), and September-November (SON). We evaluated the seasonal spatial trends of NDVI in burned areas by hemisphere, territory, or country, and by land cover types, and fire recurrences, with a focus on forested areas. The relationships between the seasonal spatial trend and three climatic variables (i.e. maximum air temperature, precipitation, and vapor pressure deficit) were then analyzed. For the 8.7 million km^2^ burned in Africa over the past 22 years, we observed several seasonal spatial trends of NDVI. The highest proportions of areas with increasing trend (*p* < 0.05) was recorded in MAM for both hemispheres, with 22.0% in the Northern Hemisphere and 17.4% in the Southern Hemisphere. In contrast, areas with decreasing trends (*p* < 0.05), showed 4.8–5.5% of burned area in the Northern Hemisphere, peaking in JJA, while the Southern Hemisphere showed a range of 7.1 to 10.9% with the highest proportion also in JJA. Regarding land cover types, 48.0% of fires occurred in forests, 24.1% in shrublands, 16.6% in agricultural fields, and 8.9% in grasslands/savannas. Consistent with the overall trend, the area exhibiting an increasing trend in NDVI values (*p* < 0.05) within forested regions had the highest proportion in MAM, with 19.9% in the Northern Hemisphere and 20.6% in the Southern Hemisphere. Conversely, the largest decreasing trend (p < 0.05) was observed in DJF in the Northern Hemisphere (2.7–2.9%) and in JJA in the Southern Hemisphere (7.2–10.4%). Seasonally, we found a high variability of regeneration trends of forested areas based on fire recurrences. In addition, we found that of the three climatic variables, increasing vapor pressure deficit values were more related to decreasing NDVI levels. These results indicate a strong component of seasonality with respect to fires, trends of vegetation increase or decrease in the different vegetation covers of the African continent, and they contribute to the understanding of climatic conditions that contribute to vegetation recovery. This information is helpful for researchers and decision makers to act on specific sites during restoration processes.

## Introduction

Fire is an essential component in ecosystem and atmospheric dynamics [1]. The amount, composition, and moisture content of fuel, the weather conditions and the presence of an ignition source create the catalytic conditions that characterizes the occurrence of fires [2]. While lightning remains the primary natural ignition source, human activity has emerged as the dominant direct and indirect source of fire ignition across many ecosystems worldwide [3]. Africa has been described as the fire continent [4]. and fires are historically associated with human activities. As a result, many ecosystems are adapted to and dependent on fire for regeneration and maintenance [5].

Centuries of increasing human and climate change have modified fire regimes, resulting in fire as an agent of ecosystem degradation [6]. Increasing human population density across Africa and the expansion of agropastoralism has altered the extent and seasonal distribution of fire [7]. In fact, between 2003 and 2019, African tropical and subtropical grasslands, savannas and scrublands together of Africa contributed most to the globally burned area [8]. Products derived from satellite data have identified the two hemispheres of Africa as having some of the highest fire recurrences worldwide [9]. The Sub-Saharan African region comprises about 70% of the global annual burned area [10, 11]. However, the loss of forest to fire across the continent appears to represent only <1% globally [12]. Despite this, in recent years, some research has reported a decreasing trend of fires in Africa [13–16], and this has been specifically associated with population growth, agricultural expansion, and land-use changes, especially in the Northern Hemisphere [13].

Across Africa, precipitation patterns are considered as determinant factors of fire occurrence [17, 18]. Rainfall is typically highly variable, the patterns of which are influenced by important climate phenomena such as La Niña, El Niño, the Atlantic Multidecadal Oscillation and the Atlantic and Indian Ocean dipoles [19, 20]. Recent climate studies based on data from 1991 to 2021, have shown a temperature increase of approximately +0.3°C per decade, but because of regional differences in precipitation trends, aridity has increased in east and southern Africa, and decreased in West Africa [20]. Regional [21] and global [22] analyses show that drier conditions can increase fire risk in wetter parts of the continent and de reverse in more arid parts (<800 mm). The increasing trend in meteorological drought episodes in most regions of Africa, especially in tropical areas may exacerbate fire risks [23]. Therefore, we expect that climate change should affect ecosystem functioning via changing fire regimes in Africa, and it is essential to get better predictive understanding of these changes, especially in tropical forest ecosystems that are not adapted to fire.

Fires can provide also a window of opportunity for action in ecosystems restoration [24]. Forest landscape restoration (FLR) has been part of the global climate agenda and a fundamental part of ambitious international targets [25], including: the Strategic Plan for Biodiversity, Aichi Biodiversity Target 15, the Bonn Challenge, the Target 5 of the New York Declaration on Forests [26], the Kunming-Montreal Global Biodiversity Framework [27], and the African Union’s Agenda 2063 [28]. The FLR concept implies an integrated approach to achieve an ecological equilibrium of a landscape and restore ecosystem services such as climate regulation [29, 30]. On the African continent progress has been made through various initiatives, such as the African Forest Landscape Restoration (AFR100), Pan-African Agenda on Ecosystem Restoration, and the African Great Green Wall [31, 32]. However, none of these initiatives have addressed fires as ecological factor, which demands a better understanding of the fire dynamics and ecosystem recovery associated with fires [33]. Mapping post-fire vegetation recovery trends in Africa is therefore essential, as it will provide insights into the long-term impacts of fire on the vegetation structure and function. This will assist in producing evidence-based restoration projects that incorporate fire. It also provides information that supports local populations livelihoods as well as biodiversity conservation strategies.

The use of modern earth observation techniques for multiscale and multitemporal monitoring across the African continent is key in filling the gap on post-fire recovery. In recent years, studies based on remote sensing products have increased in the African continent, especially to characterize to understand the vegetation phenology and its drivers. Different approaches, such as spectral indices [34] have been used to identify phenological dynamics, leaf area index or the fraction of absorbed photosynthetically active radiation [35]. Often, the Normalized Difference Vegetation Index (NDVI) or Enhanced Vegetation Index (EVI) satellite products are used in these analyses [36], complemented with field measurements. Research based on these spectral indices has been conducted to identify trends in vegetation growth or decline for the whole African continent [e.g., 37, 38] or some regions in Africa [39–41]. However, studies of vegetation dynamics in burned areas using satellite products are scarce at the continental [e.g., 42] and national levels [e.g., 43].

The aim of this study was to evaluate the seasonal spatial-temporal trends pattern of vegetation recovery in burned areas across Africa, using a temporal analysis of NDVI data between 2001 and 2020. The specific objectives of this study were to: (i) identify the seasonality of burned areas by region; (ii) analyze the seasonal spatial trend of NDVI in burned areas by region; (iii) evaluate the seasonal spatial trend of NDVI in burned areas by land cover type; and (iv) explore the relationships between these seasonal spatial-temporal trends of NDVI in burned areas with key climatic variables (precipitation, temperature, and vapor pressure deficit). Findings from this research will set the baseline information for future monitoring work as well as facilitate development of effective regeneration enhancement strategies, post-fire.

## Materials and methods

**Fig 1:**
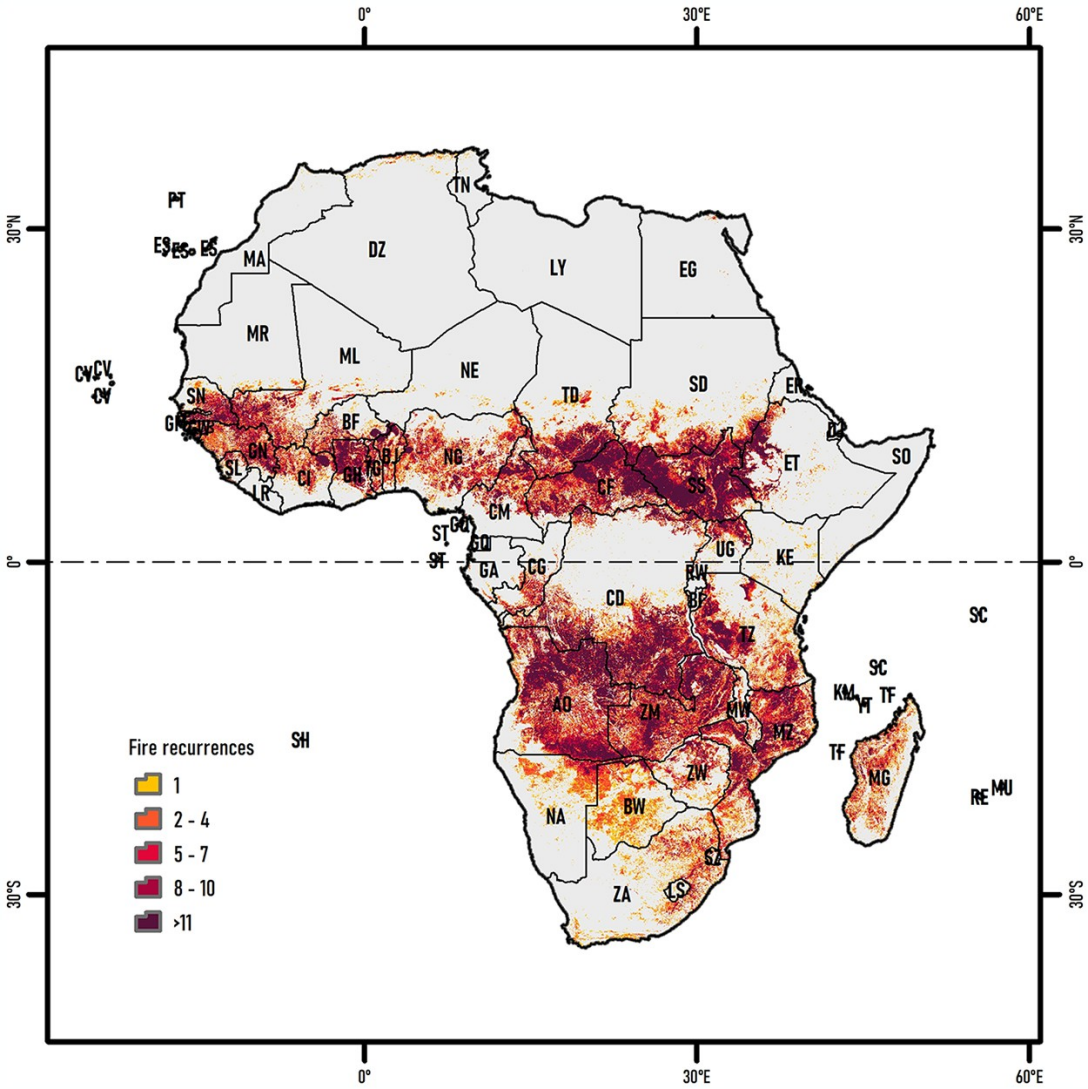
Fire recurrences in the African continent, based on the MODIS FireCCI51 product (2001–2020).

The African continent extends from 37.35°N to 34.85°S, and from 17.55°W to 51.40°E, covering an area of almost 30 million km^2^. Africa is notably asymmetrical with respect to the equator, with 20.0 million km^2^ (67%) located in the Northern Hemisphere and 9.8 million km^2^ (33%) located in the Southern Hemisphere (Fig 1). Africa is characterized by a summer wet season in which most of the precipitation falls, although the area near the equator, have two wet periods that are usually separated by a period of relatively modest precipitation [44].

The topography of Africa is diverse and consists of mountains (e.g., Mount Kilimanjaro), plateaus, plains, depressions, large coastal mini-continents (e.g., Madagascar), and small islands (e.g., Canary Islands). The combination of climatic conditions and topographical features has fostered a rich diversity of terrestrial ecosystems. Currently, 119 terrestrial ecoregions are identified across Africa and its islands, categorized into nine biomes: savanna-woodlands, deserts, tropical moist forests, montane grasslands, mediterranean scrub, flooded grasslands, tropical dry forests, mangroves, and temperate conifer forests [45, 46]. Most of these ecoregions, in both hemispheres of the continent, have recorded fires in the past two decades, with different levels of recurrences (Fig 1).

### Datasets

Three remote sensing datasets were used in this study namely burned areas (FireCCI51), vegetation index (MOD13Q1.061), and land use and land cover (CCI-LC). We also used three climate variables from the TerraClimate dataset (i.e. maximum air temperature, precipitation and vapor pressure deficit). The following sections detail these products as well as the data processing and statistical analyses.

#### Burned areas

To determine the area impacted by fires, we used the MODIS Fire_cci Burned Area pixel product version 5.1 (FireCCI51) [47, 48], a product developed by the European Space Agency’s (ESA) Climate Change Initiative (CCI), with a spatial resolution of 250 m. The FireCCI51 product is based on the red (R) and NIR (near-infrared) bands of Terra satellite data from the Moderate Resolutions Imaging Spectroradiometer (MODIS) and active fire information from thermal channels that are able to detect burnt patches [47]. FireCCI51 validation for the period 2003–2014 showed an average omission and commission errors of 67.1% and 54.4% [48]. For this study we used annual data from 2001 to 2020.

#### Normalized difference vegetation index

For this study, we used the MOD13Q1.061 Version 6.1 product [49]. MOD13Q1.061 provides two primary vegetation layers: Normalized Difference Vegetation Index (NDVI) and Enhanced Vegetation Index (EVI). These layers have a spatial resolution of 250 m generated every 16 days [49]. We used NDVI values (2001–2020), the most widely used as a proxy to quantify vegetation conditions (e.g., health, density, biomass). The NDVI data were derived from the R and NIR bands [50] of MODIS data. Values range between 1 and −1, where values close to 1 indicate abundance of photosynthetic activity and, therefore, greater growth or recovery of vegetation, while values close to −1 reflect the opposite [50].

#### Land cover type

The European Space Agency (ESA) Climate Change Initiative Land Cover (CCI-LC) developed a series of global land cover maps with yearly intervals at 300 m spatial resolution, available for the period 1992–2020 [51]. These annual classifications are derived from AVHRR (1992 to 1999), SPOT-VGT (1999 to 2013), PROBA-V (2014 to 2019), and S3-OLCI for 2020 [52]. The CCI-LC presents a classification of 22 land cover types, but for this study, we used the equivalences of nine IPCC land categories: agriculture, forest, grassland/savannas, wetland, settlement, shrubland, sparse vegetation, bare area, and water [52]. We downloaded and used the datasets for the years 2000 and 2020.

#### Climate data

The TerraClimate dataset was developed by University of Idaho using a combination of high-spatial-resolution climatological from the WorldClim dataset, the time-varying data from CRU Ts4.0, and the Japanese 55-year Reanalysis by using climatically aided interpolation [53]. TerraClimate has a spatial resolution of ~4 km and covers the monthly and annual period from 1958 to the near-present [53]. The maximum air temperature (Tmax, °C) and precipitation (P, mm) have been widely used to assess vegetation growth in Africa [e.g., 54–56], but the vapor pressure deficit (VPD) is one of the most relevant surface meteorological variables for plant physiology [57]. For this study we used the monthly values for 20 years (2001–2020) of Tmax, P and VPD data.

#### Data processing and statistical analyses

Using the cloud computing platform Google Earth Engine (GEE) [58], we developed scripts to annually identify burned areas, fire recurrences, NDVI time series and climate variables, between 2001 and 2020. The data was separated into the four standard meteorological seasons: December-February (DJF), March-May (MAM), June-August (JJA) and September-November (SON). This quarterly stratification was performed in order to identify patterns of seasonal trends of NDVI values for the two hemispheres, due to the high monthly and annual climatic variability of Africa [20]. We detected vegetation trends over the 20-year time period independently for the four three-month seasonal periods (DJF, MAM, JJA and SON), using the Mann-Kendall test statistic [59]. This non-parametric test is mainly used to determine statistically significant monotonic trends [60]. Analyses were performed with the entire available dataset of NDVI pixels from the same seasons in areas that burned in at least a single fire per year, including pixels where no previous fires were recorded, and were not limited to identifying post-fire trends from a specific year. Over the 20-year period, each pair of three-month periods was compared, obtaining the Mann-Kendall statistic with the associated trend *Z*-scores and *p*-values for each pixel. The result was +1 if the *Z*-score at the second time step was greater than the *Z*-score at the first time step, −1 if the opposite is true, and zero if they were both equal. This statistic accounts for the possible effect of autocorrelation on trend detection by returning the corrected *p* values after accounting for temporal pseudoreplication [61]. To facilitate a direct comparison between different regions worldwide, we presented results using three categories of trends: increasing (positive) and significant (*p* < 0.05), decreasing (negative) and significant (*p* < 0.05) and non-significant (*p* > 0.05). We then intersected the annual burned areas with the results of the seasonal trends of NDVI values (DJF, MAM, JJA, SON), for the whole continent, two hemispheres, countries or territories. Additionally, in order to identify changes between periods and trends of cover increase and decrease, we intersected the 2000 and 2020 land cover type maps with the burned area measured over 20 years, focusing on forested areas within the study domain. We then evaluated the trends of increase or decrease of NDVI values for the different land cover types using the year 2020 as a reference. Finally, we identified forest area regeneration trends based on wildfire recurrence.

We evaluated the relationships between these seasonal spatial-temporal trends of NDVI values (dependent variable) in burned areas and key climatic variables (independent variables) using the Pearson correlation coefficient (*r*). The coefficients range from −1 to 1 and represents the strength of the linear association between the vegetation and climatic factors [62]. For our study, a value of *r* above 0.8 (positive) and below −0.8 (negative) indicated a strong linear correlation; an *r* between 0.4 and 0.8 (positive), and −0.8–−0.4 (negative) implied a moderate linear correlation; and an *r* between −0.4 and 0.4 (except *r* = 0) was considered a weak linear correlation. In GEE, we developed a mask using the results of the three trend categories and performed a random distribution of points for the entire quarterly data series of maximum air temperature, precipitation, and vapor pressure deficit.

## Results

### Seasonality of burned areas by region

**Fig 2:**
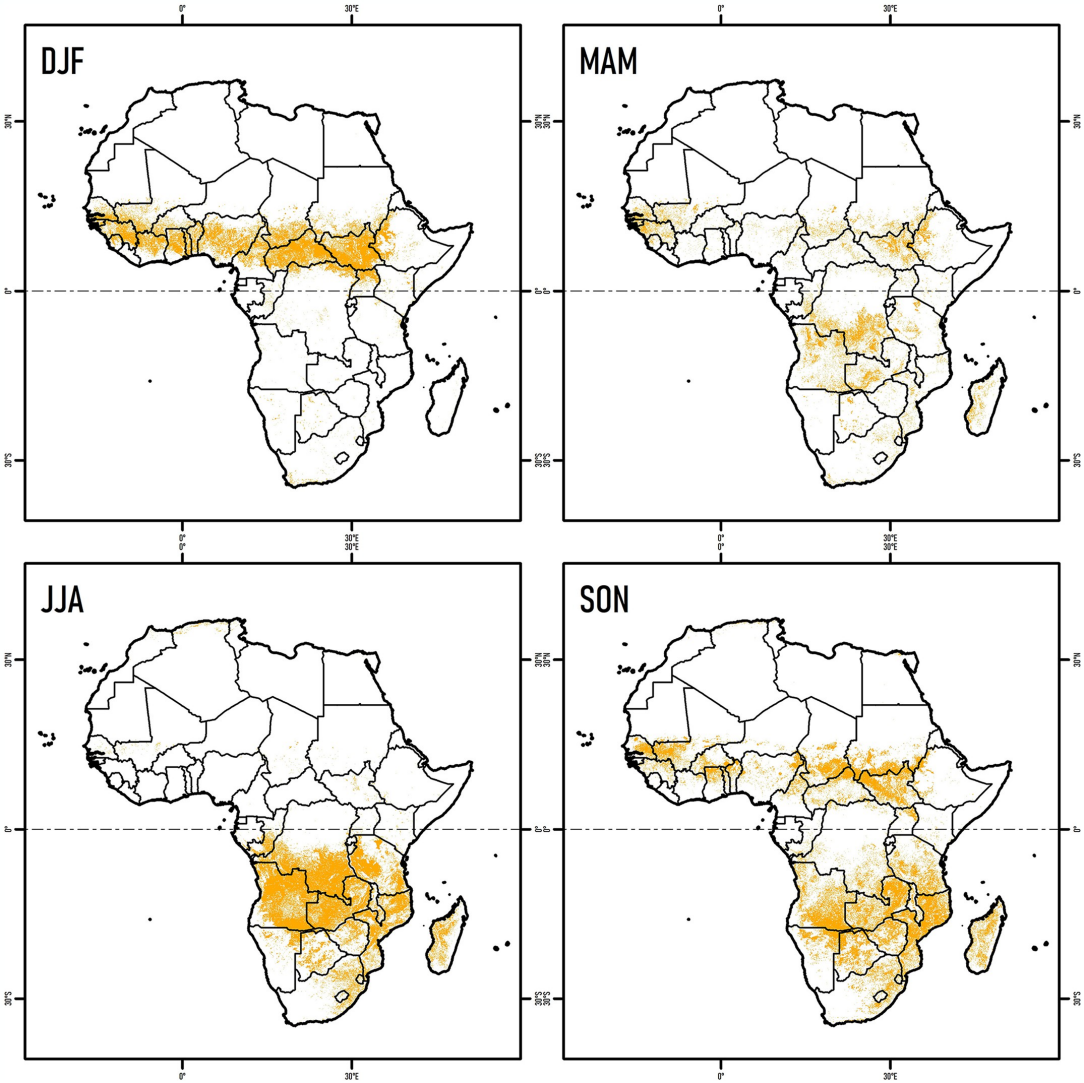
Seasonality of burned areas (yellow) in Africa (2001–2020) for DJF (December-February), MAM (March-May), JJA (June-August) and SON (September-November).

At the continental scale (including islands), the absolute area of fires in the period 2001–2020 was ca. 8.7 million km^2^, representing 29.2% of the entire African territory (Fig 2). The average series shows that fires occurred in 53 of 61 countries or territories during the 20 years study period, of which the Democratic Republic of the Congo (983,316 km^2^), Angola (953,123 km^2^), Zambia (650,969 km^2^), Mozambique (577,992 km^2^), South Sudan (576,624 km^2^), Central African Republic (510,636 km^2^), Tanzania (472,057 km^2^), Nigeria (419,132 km^2^), Chad (310,857 km^2^) and Sudan (302,634 km^2^), were among the ten hotspots with the largest mean burned areas. Of these countries, South Sudan (91.1%), Zambia (86.6%), Central African Republic (82.5%), Guinea (78.5%), Angola (76.45%), Togo (75.1%), Mozambique (73.5%), Benin (71.4%) and Ghana (60.9%), presented the highest proportions of burned areas in relation to the national surface. These results indicate that most of the countries or territories in Africa have recorded fire events in the last two decades, and that in some of these, fire has burned more than half of their land area.

**Fig 3:**
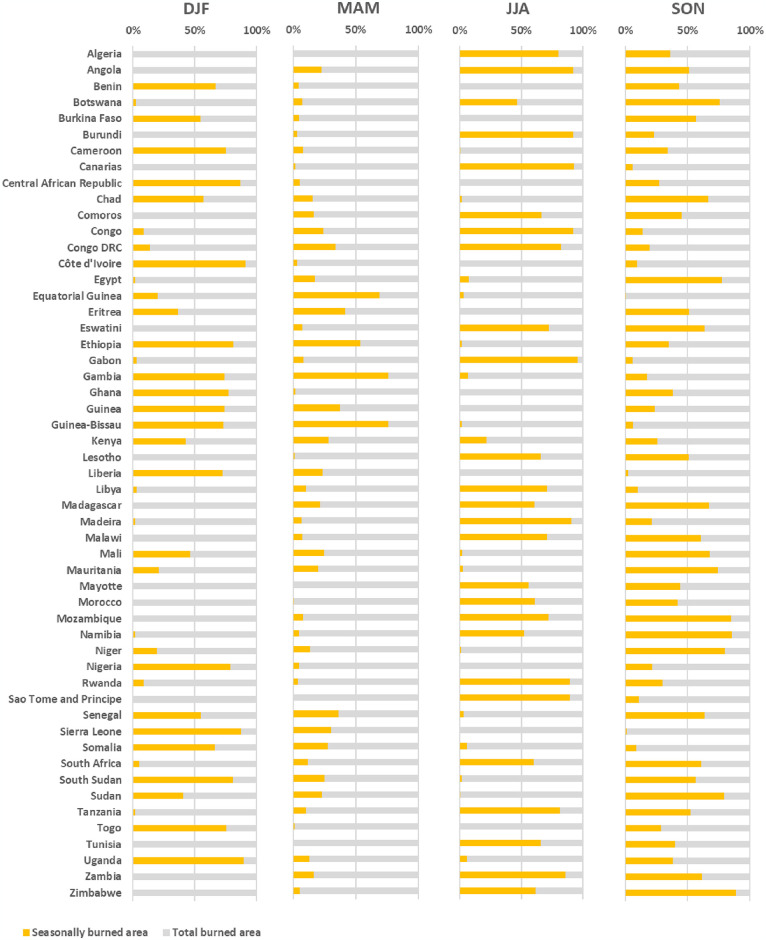
Seasonality of burned areas (yellow) by countries and territories (2001–2020). DJF (December-February), MAM (March-May), JJA (June-August), and SON (September-November).

Overall, our analyses showed that there was a marked seasonal and spatial pattern of fires across the continent. In the Northern Hemisphere, fires were mainly concentrated in DJF (97.6%), but decrease in MAM (43.1%) and were almost completely absent in JJA (1.5%). This timing is in contrast with the Southern Hemisphere, which exhibited an increase of 98.5% for JJA, and then an increase in SON (37.7%) (Fig 2 and Table 1). In addition, the series show that proportionally, the countries or territories with the largest seasonally burned areas were Côte d’Ivoire (91%), Uganda (90%) and Sierra Leone (88%), for the DJF season; Guinea-Bissau (76%), Gambia (76%), and Equatorial Guinea (69%), Gambia (76%), and Equatorial Guinea (69%), for MAM; Gabon (96%), Canary Islands (93%), and Burundi (92%) for JJA; and Zimbabwe (89%), Namibia (86%), and Mozambique (85%) for SON (Fig 3). These results highlight the wide seasonal variation in fire patterns over last two decades, and that the highest peaks were recorded in DJF and JJA, as was seen across numerous different countries or territories.

**Table 1:** Seasonality (%) of burned areas across Africa (2001–2020) for the two hemispheres. DJF (December-February), MAM (March-May), JJA (June-August), and SON (September-November).

Seasons	Northern Hemisphere (%)	Southern Hemisphere (%)	Total
DJF	97.6	2.4	100
MAM	43.1	56.9	100
JJA	1.5	98.5	100
SON	37.7	62.3	100

#### Seasonal spatial trend of NDVI in burned areas by region

**Fig 4:**
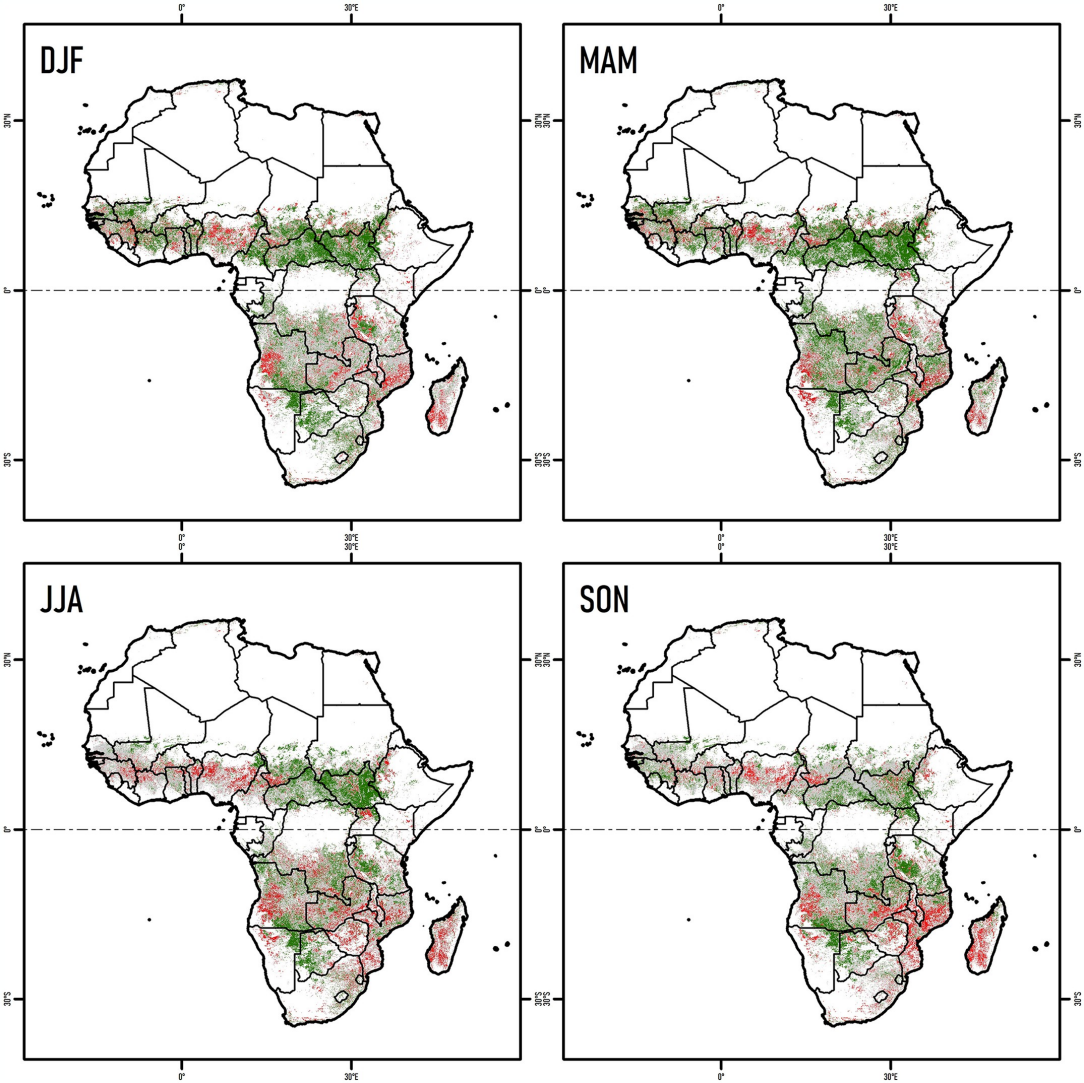
Seasonal spatial-temporal trend of NDVI in burned areas across Africa (2001–2020). Areas with increasing (green) or decreasing (red) vegetation, based on the Mann-Kendall test and statistically significance values (*p* < 0.05) for DJF (December-February), MAM (March-May), JJA (June-August), and SON (September-November). Gray areas were not statistically significant (*p* > 0.05).

The trends of NDVI over 20 years of fire events in Africa exhibited diverse spatial variations. In the Northern Hemisphere 22.0% of the area showed an increasing NDVI trend in MAM, and 20.4% showed an increasing trend in DJF (Table 2 and Fig 4). In the Southern Hemisphere, only 17.4% recorded an increasing trend in MAM, while the rest of the seasons presented values ranging from 13.2 to 13.8% (Table 2 and Fig 4). Areas showing decreasing trends (*p* < 0.05) in the Northern Hemisphere, ranged from 4.8–5.5% with strongest decreases in JJA. In the Southern Hemisphere however, the area of decreasing trends ranged from 7.1–10.9% with the strongest decreases also occurring in JJA (Table 2 and Fig 4). Importantly, in both the Northern and Southern Hemispheres, over half of the land area showed no NDVI trend (p > 0.05) values ranged from 48.7–60.8%) (Table 2 and Fig 4). These results indicated that even without considering the type of vegetation, the patterns of NDVI trends were not proportionally related to the marked seasonality of the burned areas, and the trends of NDVI increase were higher than the trends of decrease.

**Table 2:** Seasonal spatial-temporal trend of NDVI in burned areas across Africa (2001–2020). Percentages (%) of increases or decreases (%) based on the Mann-Kendall test and statistically significant (*p* < 0.05) and non-significant (p > 0.05) values. DJF (December-February), MAM (March-May), JJA (June-August), and SON (September-November).

Seasons	Northern Hemisphere (Increasing, *p* < 0.05)	Southern Hemisphere (Increasing, *p* < 0.05)	Northern Hemisphere (Decreasing, *p* < 0.05)	Southern Hemisphere (Decreasing, *p* < 0.05)	Non-significant (*p* > 0.05)	Total
DJF	20.4	13.2	4.8	7.1	54.4	100
MAM	22.0	17.4	4.8	7.2	48.7	100
JJA	13.4	13.4	5.5	10.9	56.9	100
SON	11.1	13.8	4.3	10.1	60.8	100

**Fig 5:**
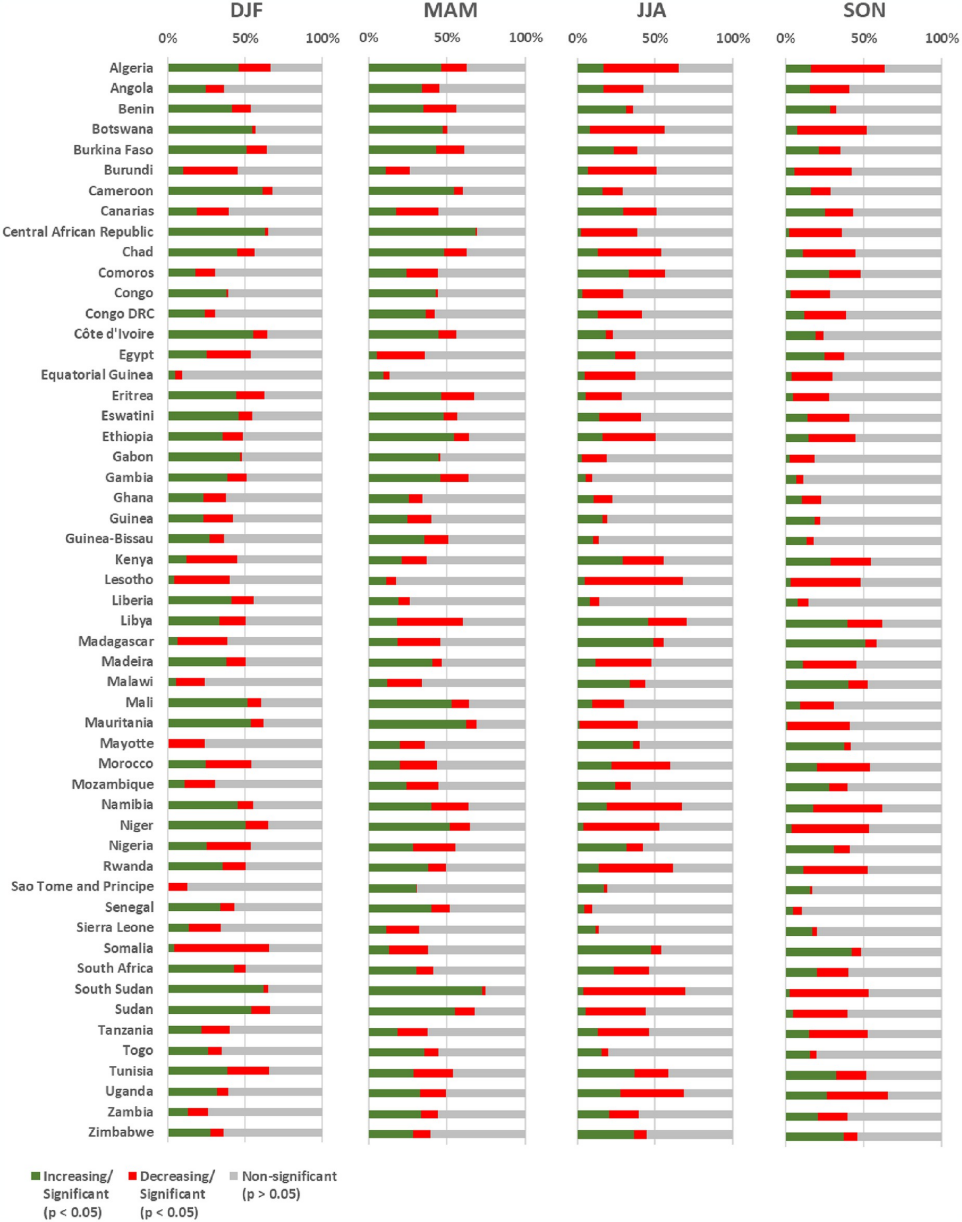
Seasonal spatial-temporal trends of NDVI in burned areas across Africa, by countries and territories (2001–2020). Analysis based on the Mann-Kendall test and statistically significance values (*p* < 0.05, *p* > 0.05) for DJF (December-February), MAM (March-May), JJA (June-August) and SON (September-November).

Fig 5 reveals a wide variability of trends in NDVI values between the four seasonal periods for most countries or territories. The seasonal differences reach up to 61% (e.g., Central African Republic) in significant increasing trends (*p* < 0.05) and up to 63% (e.g., South Sudan) among areas with decreasing trends. However, some countries or territories indicated a higher proportion of areas with significant increasing and decreasing trends (*p* < 0.05) in NDVI values (Fig 5). Seasonally, the highest proportions of increasing trends (*p* < 0.05) were recorded for Central African Republic (63%) in DJF, South Sudan (72%) in MAM, Madagascar (49%) in JJA, and Uganda (58%) in SON (Fig 5). In terms of decreasing trends, the highest proportions at country or territory level were recorded in Somalia (62%) in DJF, Libya (42%) in MAM, South Sudan (66%) in JJA, and Madagascar (50%) in SON (Fig 5).

#### Seasonal spatial-temporal trend of NDVI in burned areas by land cover type

Forty eight percent of the burned areas (2001–2020) occurred in forests, 24.1% in shrublands, 16.6% in agricultural fields, 8.9% in grasslands, and the remaining distributed in smaller proportions among the other types of coverage (wetland, settlement, sparse vegetation, and bare area). However, the results showed that in the areas burned, forests increased by 1.4% while agriculture increased in the burned areas by 0.2%. In addition, scrublands decreased by 1.6% and grasslands by 0.1%.

#### Land cover type

**Fig 6:**
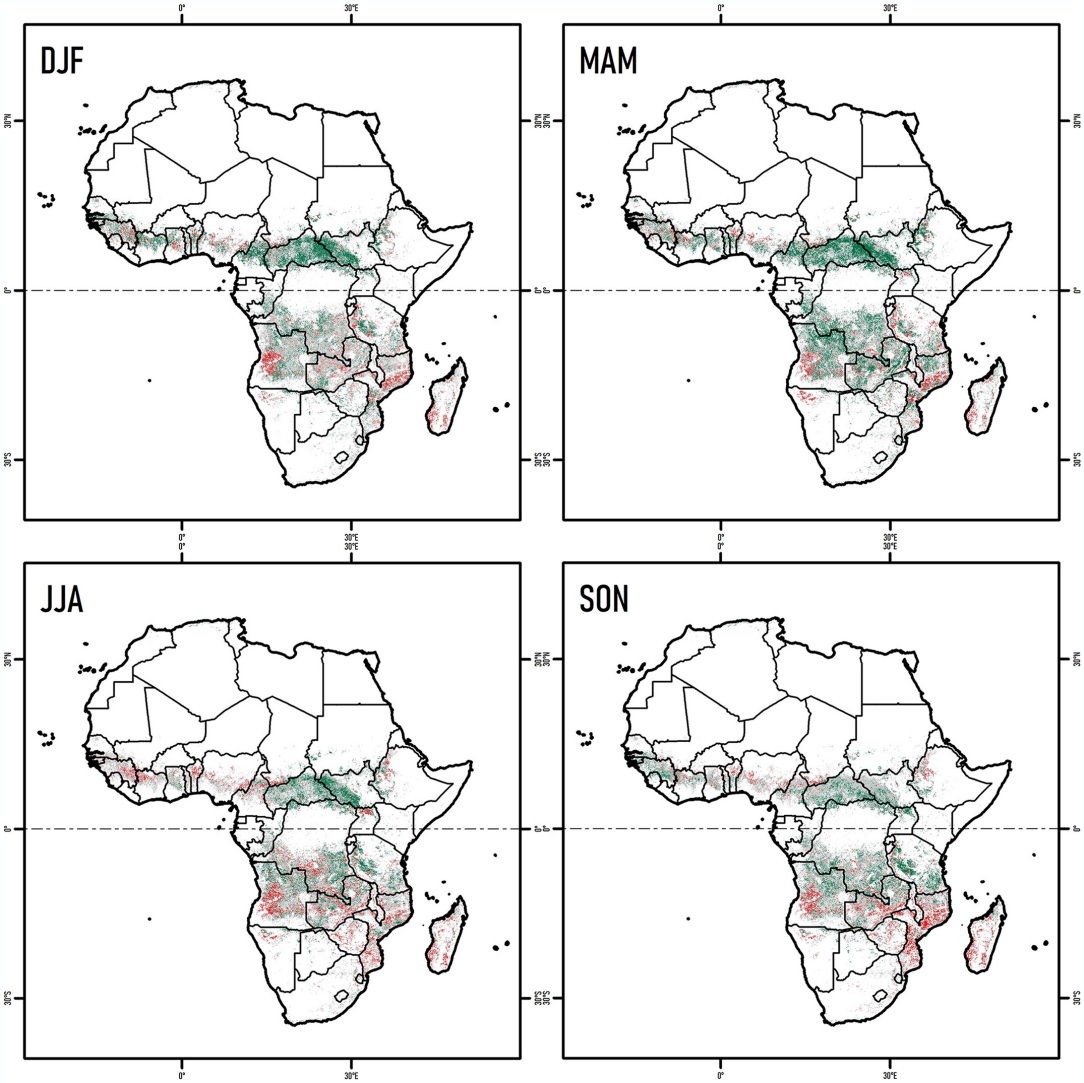
Seasonal spatial-temporal trend of NDVI of burned forested areas in Africa (2001–2020). Areas with increasing (green) or decreasing (red) vegetation, based on the Mann-Kendall test and statistically significance values (*p* < 0.05) for DJF (December-February), MAM (March-May), JJA (June-August) and SON (September-November). Gray areas were not statistically significant (*p* > 0.05).

Regarding forested areas, the seasonal spatial trend of NDVI in burned areas over 20 years in Africa exhibited diverse spatial variations. Proportionally, the highest peak of increasing trend of NDVI (*p* < 0.05) of forested areas in the Northern Hemisphere was recorded in MAM (19.9%), followed by DJF (18.9%) (Table 3 and Fig 6). In the Southern Hemisphere, the highest peak of increasing area (*p* < 0.05) was recorded at MAM (20.6%), while the other seasons measured values ranged from 11.9–13.8% (Table 3 and Fig 6). As for the decreasing trends of forested areas (*p* < 0.05), 2.7–2.9% of the area of the African Northern Hemisphere was measured, with the highest peak of decrease occurring in DJF (Table 3 and Fig 6). In the Southern Hemisphere, the area of decreasing trends ranged from 7.2–10.4% with the highest peak of decrease occurring in JJA (Table 3 and Fig 6). However, the highest proportion of NDVI of forested areas in all seasons (49.6–63.2%) was represented by non-statistically significant values (*p* > 0.05) (Table 3 and Fig 6). In general, the results showed that until 2020, forested regions with an increasing trend in NDVI values represent a surface of 211,026 km^2^, while forested areas with decreasing trend were 169,899 km^2^.

**Table 3:** Seasonal spatial-temporal trend of NDVI of burned forested areas in Africa (2001–2020) for the two hemispheres. Percentages (%) of increases or decreases (%) based on the Mann-Kendall test and statistically significance (*p* < 0.05) and non-significant (*p* > 0.05) values. DJF (December-February), MAM (March-May), JJA (June-August), and SON (September-November).

Seasons	Northern Hemisphere (Increasing, *p* < 0.05)	Southern Hemisphere (Increasing, *p* < 0.05)	Northern Hemisphere (Decreasing, *p* < 0.05)	Southern Hemisphere (Decreasing, *p* < 0.05)	Non-significant (*p* > 0.05)	Total
DJF	18.9	11.9	2.9	7.6	58.8	100
MAM	19.9	20.6	2.7	7.2	49.6	100
JJA	10.7	12.9	2.7	12.7	61.0	100
SON	9.9	13.8	2.7	10.4	63.2	100

**Fig 7:**
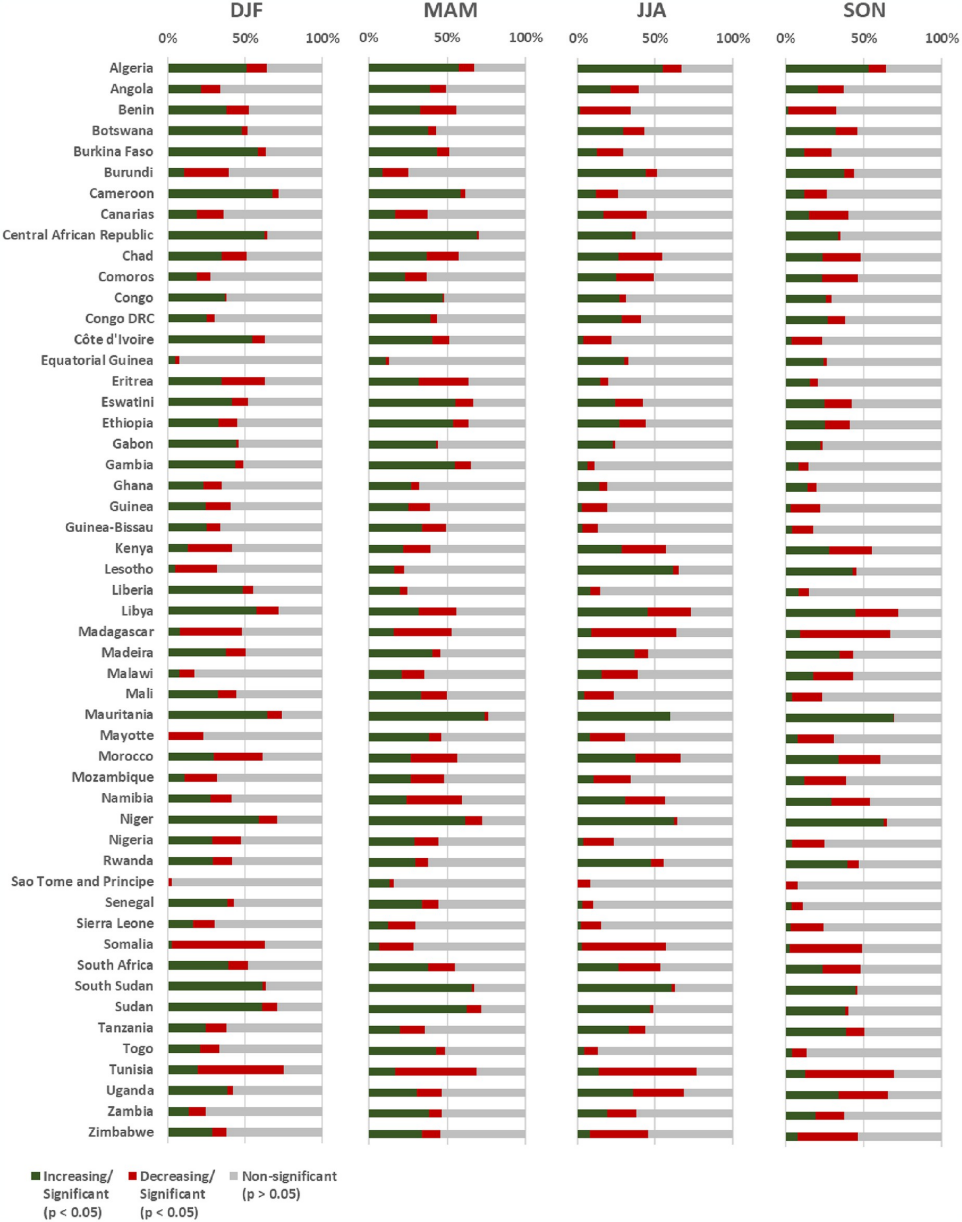
Seasonal spatial-temporal trend of NDVI of burned forested areas in Africa (2001–2020) by countries and territories. Areas with increasing (green) or decreasing (red) vegetation, based on the Mann-Kendall test and statistically significance values (*p* < 0.05) for December-February (DJF), March-May (MAM), June-August (JJA) and September-November (SON). Gray areas were not statistically significant (*p* > 0.05).

Fig 7 shows a large variability of trends of NDVI values in forested areas between the four seasonal periods (DJF, MAM, JJA, SON) for most countries or territories. However, some countries or territories present a higher proportion of areas with significant increasing and decreasing trends (*p* < 0.05) in NDVI values (Fig 7). Seasonally, the highest proportions of increasing trends (*p* < 0.05) of forested areas were recorded for Cameroon (68%) in DJF, Mauritania in MAM (74%) and SON (72%), and Niger (62%) in MAM (Fig 7). In terms of decreasing trends, the highest proportions at country or territory level were recorded in Somalia (60%) for DJF, Tunisia for MAM (52%) and JJA (63%), and Madagascar (56%) for SON (Fig 7).

Overall, the annual averages show that the countries or territories with the highest statistically significant growth trends (*p* < 0.05) in areas identified as forest ecosystems were Mauritania with 68% (±6.8), Niger with 61% (±1.4), Algeria with 54% (±2.6), South Sudan with 53% (±19.2), Central African Republic with 49% (±19.4). In contrast, statistically significant decreasing trends were found in Tunisia with 57% (±4.8), Madagascar with 47% (±10.0), Somalia with 44% (±17.3), Morocco with 32% (±3.5) and Mozambique with 26% (±8.2).

#### Regeneration trends of forested areas based on fire recurrences

The results show a wide variability of fire recurrences in 20 years. In general, fire recurrences are spatially represented mainly by sites that burned only once (18.1%), 2 times (10.9%), 3 times (8.1%), followed by decreasing percentages by increasing recurrences numbers. For forested areas, the pattern was found to be similar, but sites that burned only once had a proportion of 11.2%. Some forested areas burned up to 25 times (MAM, SON) and in other seasons reached a maximum of 32 (JJA) and 35 times (DJF). This indicates that in certain regions, fires may occur more than once per year.

**Fig 8:**
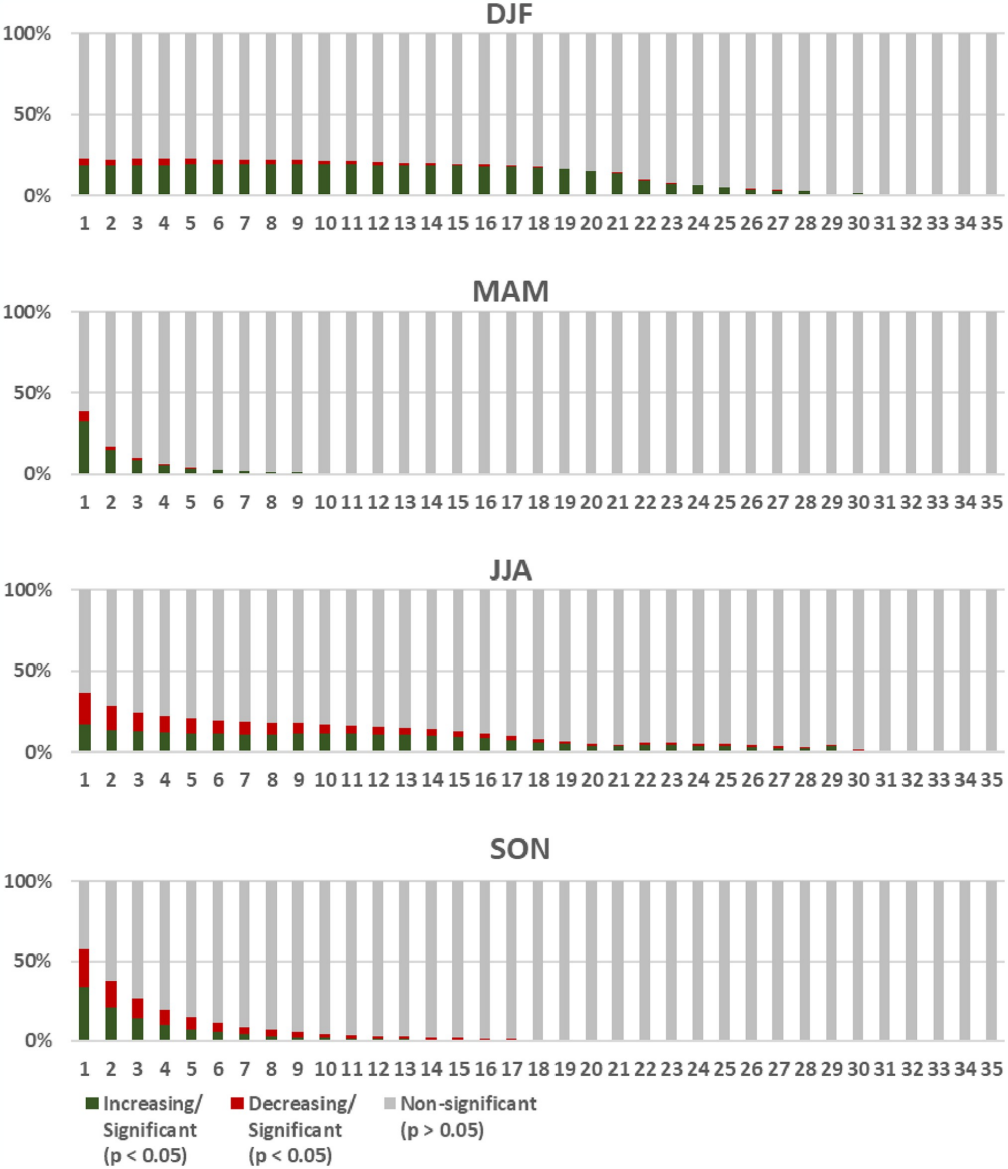
Proportion of the increase (green) or decrease (red) in forest cover in areas with different fire recurrences in Africa, according to the Mann-Kendall test and statistically significance values. DJF (December-February), MAM (March-May), JJA (June-August), and SON (September-November). Gray areas were not statistically significant (p > 0.05).

Seasonally, we found a high variability of regeneration trends of forested areas based on fire recurrences. In DJF, areas with statistically significant vegetation growth trends (*p* < 0.05) were higher in sites that burned at least 20 times, whereas in MAM this pattern was evident in fire recurrences with total of 1–2 fires. However, in JJA, statistically significant decreasing trends were highest in fire recurrences of between 1–5 times, subsequently exhibiting a reversal of the trend pattern that was seen in other seasons. For SON, it was observed that from 6–13 fire recurrences, the trends from increasing to decreasing were also reversed (Fig 8). These results demonstrate that, although seasonal fire recurrences may reflect differences in fire regimes in Africa, for areas that showed significant trends, it is evident that forests that burn less frequently in some seasons (MAM, SON) were more likely to recover.

#### Relationships between spatial-temporal trend of NDVI with climatic variables in burned areas

**Fig 9:**
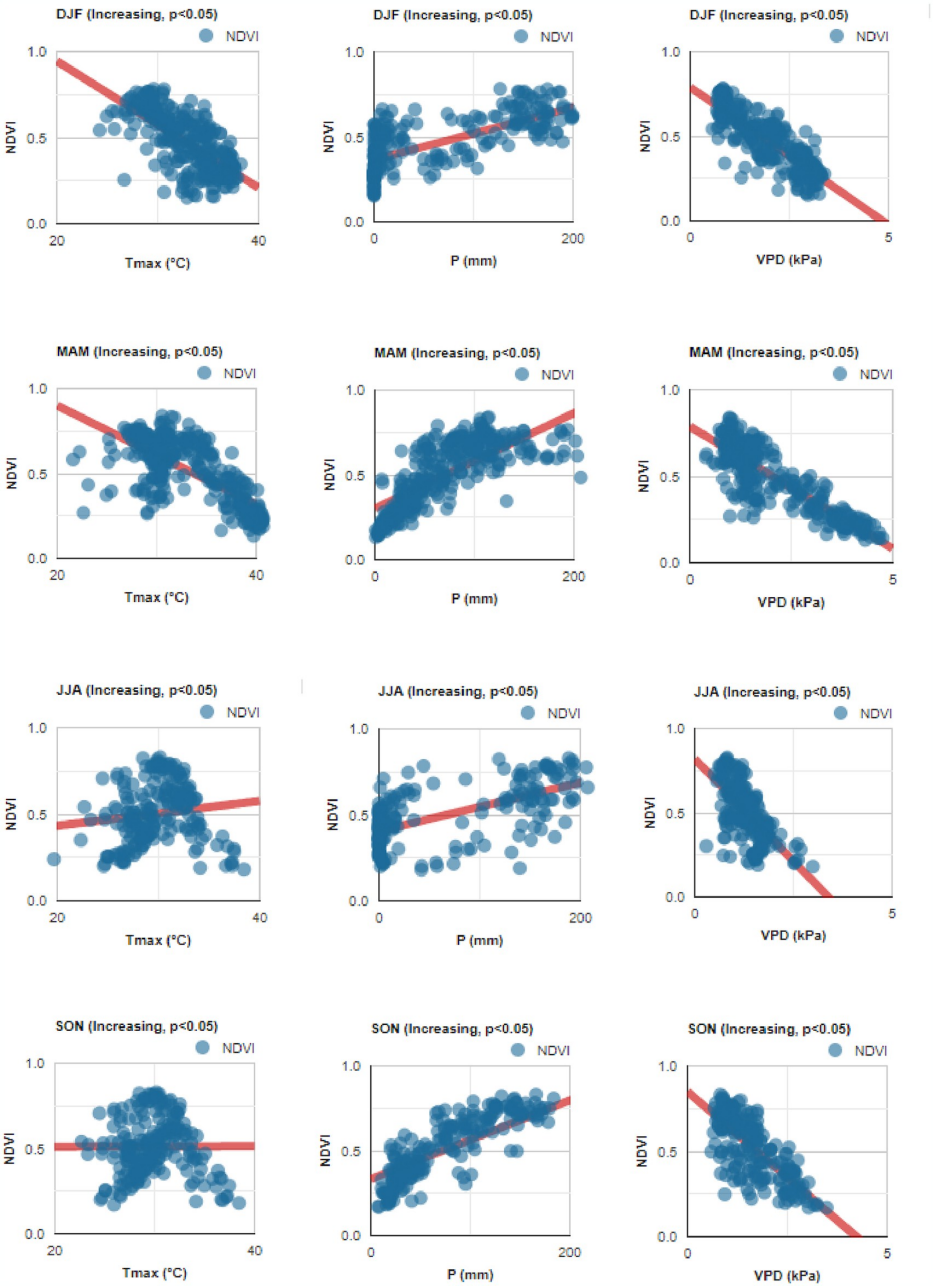
Relationship between monthly NDVI and climatic variables values (Tmax, maximum air temperature; P, precipitation; VPD, vapor pressure deficit), in areas with increasing trends (*p* < 0.05). DJF (December-February), MAM (March-May), JJA (June-August), and SON (September-November).

**Fig 10:**
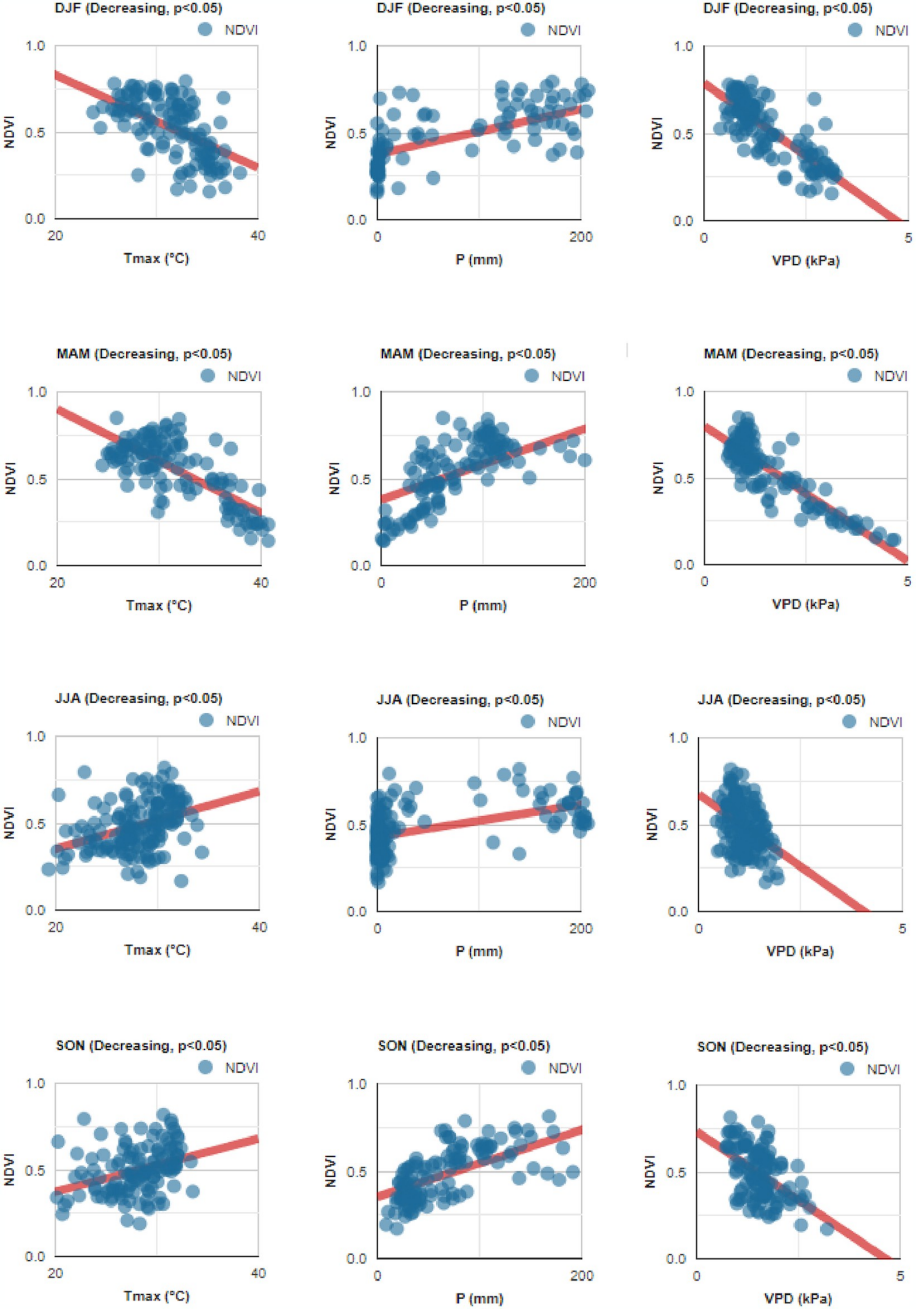
Relationship between monthly NDVI and climatic variables values (Tmax, maximum air temperature; P, precipitation; VPD, vapor pressure deficit), in areas with decreasing trends (*p* < 0.05). DJF (December-February), MAM (March-May), JJA (June-August), and SON (September-November).

Seasonally, not all climatic variables were related to increasing or decreasing trends in NDVI values. In the case of maximum air temperature (Tmax), the seasons DJF and MAM showed a moderate negative relationship in both increasing (*r* = −0.560, *r* = −0.707) and decreasing trends (*r* = −0.680, *r* = −0.754). It was observed that the lower the NDVI value, the higher the Tmax (Table 4 and Figs 9 and 10). In DJF and MAM, as Tmax increased the NDVI value decreased, but the opposite occurred in JJA and SON, both in areas with increasing and decreasing trends. For precipitation (P) values, the four seasonal time periods showed moderate positive relationships, especially for increasing trends at DJF (*r* = 0.746), MAM (*r* = 0.740) and SON (*r* = 0.777) (Table 4 and Figs 9 and 10). The NDVI index increased in relation to higher P, both in sites with increasing and decreasing trends. However, the vapor pressure deficit (VPD) presented a strong negative relationship (Table 4 and Figs 9 and 10), especially in the DJF season for increasing (*r* = −0.850) and decreasing trends (*r* = −0.803), MAM for increasing (*r* = −0.860) and decreasing trend (*r* = −0.870). In all four periods, NDVI values decreased when the VPD increased. These findings highlight the complex interactions between climate and vegetation dynamics across different seasonal periods. While increasing Tmax (two seasonal periods) and VPD values (all periods) reduced vegetation growth, increasing P values (two seasonal periods) promoted vegetation growth.

**Table 4:** The correlation coefficient (*r*) between NDVI values and the seasonal series (2001–2020) of maximum air temperature (Tmax), precipitation (P), and vapor pressure deficit (VPD). DJF (December-February), MAM (March-May), JJA (June-August), and SON (September-November).

Seasons	Trend	Tmax (°C)	P (mm)	VPD (kPa)
DJF	Increasing	−0.560	0.746	−0.850
DJF	Decreasing	−0.680	0.729	−0.803
MAM	Increasing	−0.707	0.740	−0.860
MAM	Decreasing	−0.754	0.629	−0.870
JJA	Increasing	0.149	0.637	−0.653
JJA	Decreasing	0.368	0.574	−0.387
SON	Increasing	0.003	0.777	−0.750
SON	Decreasing	0.352	0.639	−0.506

## Discussion

### Seasonality of burned areas by region

This study analyzed the recent seasonal spatial trends of vegetation recovery in burned areas across Africa, focusing on understanding the factors influencing recovery and fire regimes. A first analysis allowed us to identify the spatial and temporal distribution of burned areas, as well as seasonal variations in wildfire regimes. An important aspect to consider in satellite monitoring is spatial resolution. Recent studies using MODIS 500 m products indicate that burned area in Africa declined by 18.5% (2002 to 2016), with the strongest decline in the Northern Hemisphere [15], while for the Central African region (2003–2017), a decrease in area burned from 27,000–32,000 km^2^ per year has been reported [63]. Evidence from sub-Saharan Africa suggests that burned area decreased at a rate of ~36,000 km^2^ per year (2001–2020) [16]. In this study, using a 250 m MODIS product (FireCCI51), we found that the total absolute area of burned areas was 8.7 million km^2^ (2001–2020). Nevertheless, it has been demonstrated that values using medium resolution products (e.g., MODIS) may underestimate the area burned [15]. Some analyses of burned areas using high-resolution satellite products (Sentinel-2) suggest that the burned area is much larger than previously estimated, by as much as 50% [64]. In this sense, future studies to estimate spatial regeneration trends should use Sentinel-2 imagery, with at least ten years of data, to avoid statistical errors in the Mann-Kendall test.

We found that fires were recorded in 87% of the countries or territories across the continent. The Democratic Republic of Congo, the country with the largest burned area, traditionally practices shifting cultivation [65] which is also the main cause of deforestation [66]. As with many African countries, this practice consists of cutting down the primary forest, letting the resulting woody residue dry, collecting part of it as firewood, and then burning the remaining vegetation after several weeks or months. The area is then used for agricultural activities [67, 68]. In the case of Angola, the country with the second largest burned area, fire has played an important role in shaping the landscape, particularly within the dominant Miombo wet savanna biome [69].

Fires in Africa have been described as seasonally occurring, in the Northern Hemisphere mainly from October to March, with a peak in December to January; and in the Southern Hemisphere from April to October, with a peak in August [70, 71]. In this study, we found a similar seasonal pattern using the FireCCI51 product for the 20 years of analysis (2001–2020) [9]. Additionally, in the Northern Hemisphere, fires are mainly concentrated in DJF (97.6%). However, this trend was reversed in the Southern Hemisphere in JJA (98.5%). The high frequency of changes in these fires during the dry season in both hemispheres can be explained by the availability of dry biomass fuel, owing to the lower precipitation and atmospheric humidity, and to higher temperatures. However, recent studies have shown that fires in the Northern Hemisphere of Africa are also sensitive to the sea surface temperature variability of tropical oceans, especially those in the tropical Atlantic Ocean and tropical Indian Ocean, while in the Southern Hemisphere, Atlantic Ocean temperature exerts a relatively strong control on fires in the southern region of the continent [72].

### Seasonal spatial-temporal trend of NDVI in burned areas by region

The spectral indices derived from satellite products, such as the Normalized Difference Vegetation Index (NDVI) or the Enhanced Vegetation Index (EVI), have been fundamental in monitoring vegetation dynamics and change [73]. Spectral indices are characterized by varying sensitivity to different vegetation properties. Particularly the NDVI, which is derived from the optical reflectance of sunlight in the red and near-infrared wavelengths, is highly responsive to changes in vegetation conditions. The NDVI values increase response to vegetation greening and decreases when vegetation undergoes browning, thus serving as an effective indicator of vegetation health [34]. In addition, high NDVI values are related to dense vegetation with high near-infrared band reflectivity values. However, results obtained using spectral indices should be interpreted with caution as there may be confusion between the greenness detected for grasses and the forest canopy, as is the case for using NDVI reported in South Africa [74] and EVI in Angola [75].

In recent decades, there has been an increase in the publication of studies based on spectral indices to determine phenology patterns on the African continent [35]. Some of these investigations using MODIS products, found an increasing trend of vegetation in the Sahel region and a decreasing trend in the Central African region (1982–2015) [56]. Bimodal phenological patterns have also been described in East Africa, specifically in the Horn of Africa, and in some parts of West Africa, particularly on the Guinea coast [37, 76]. Phenological parameters such as early season and late season have been studied using the MODIS EVI product (2001–2012) and found that latitude had more influence on SOS and EOS in the Northern Hemisphere relative to the Southern Hemisphere [76]. This same pattern was found in another recent study using NDVI (1982–2015) and included the delays in the peak growing season [38]. In addition, it has been estimated that 2.1% of the African continent experienced local greening, especially in semi-arid environments (2001–2021) [36]. However, the identification of vegetation regeneration trends in burned areas using NDVI products are apparently scarce [42].

The African continent has highly spatially irregular and multi-year temporally variable vegetation phenology patterns [40, 76]. It is for this reason that our results showed that there were seasonally variable vegetation regeneration trends by region, but this trend variability was not found to be associated with the seasonality of fire events in the two hemispheres. The highest peak of the proportions of these areas with increase trends of NDVI was recorded in the MAM, while the decreasing trend peak was recorded in the JJA for both hemispheres.

A limitation of this study was the pixel resolution of the products analyzed; future studies using higher resolution datasets, such as Landsat or Sentinel-2, with similarly longer time series would improve the ability to quantify and identify small forest areas, and more accurately determine the trends of vegetation increase or decrease through time. In addition, due to the cross-continent study area, it was not possible to provide ample validation with in situ data. Our, analysis could be improved with *in situ* vegetation cover data, particularly to provide a better understanding of the spectral differentiation between woody canopy and herbaceous cover, for improved recovery estimation. Future studies should focus on using field data in combination with estimates based on satellite products.

### Seasonal spatial-temporal trend of NDVI in burned areas by land cover type

Savannas are dynamic ecosystems with a diverse mix of grasses and trees, that may vary across time and space [77]. The African continent has the largest area of grasslands and savannas, covering about one-third of the land surface [78], mainly in the Southern Hemisphere [79]. In both the Southern Hemisphere (90%) and Northern Hemisphere (82%) savanna fires were the most dominant across Africa, followed by grasslands, shrublands, forests, and croplands (1996–2012) [12]. Some studies found savannas, grasslands, and croplands, respectively notably decreasing trends in fire (2001–2020), while forests have shown a significant increasing trend within the same period [16]. In this study we identified that grasslands/savannas represent only 8.9% of the burned areas, surpassed by shrublands, agricultural lands, and forests. From an ecological perspective, grasslands and savannas are considered fire-dependent, as plant and animal species show various adaptations and interactions [80–82]. In addition, the exclusion of fire in some habitats had resulted in the spread of shrublands and forests [83, 84]. Grasslands resprout quickly after a fire, which makes this ecosystem prone to more frequent fires [17, 69]. On-ground fire regimes, often triggers seed germination, flowering and seed dispersal in fire-dependent plant communities [4]. In this sense, prescribed fire (burns) could be fundamental in the conservation and restoration of savannas and grassland biodiversity [85]. In shrublands, perennial woody shrub species generally regenerate after fire by reseeding from seed, but also through resprouting, a strategy that allows plants to survive fire from surviving underground structures [86].

Furthermore, Africa is home to 16% of the world’s forests [87], including the Congo Basin’s Forest, the second largest rainforest on the planet [88]. However, deforestation rates in Africa are alarming [68, 87]. Between 2010 and 2020, the highest annual rate of net forest loss was recorded at 39,000 km^2^ [87]. Decreasing trends between the fraction of forest loss and fires have been found in areas with relatively low tree cover. An increasing trend of forest loss (2001–2019) was found in all African countries, due to fires ≥10km^2^ per year [89]. In addition, increasing trends in forest loss and active fire detection have been observed in areas with high tree cover, such as the Congo Basin [90]. In fact, 78% of the areas burned in forested areas globally were in Africa (2001–2019) [87], representing a loss of 2% area due to fires [91]. In our study, results indicated that at the land cover type level, forests spatially represented almost half of the burned areas of the entire continent in the last two decades. Despite different seasonal fire patterns between the two hemispheres, overall we found a high rate of forest recovery. However, we also noted that during the time the DJF period when the highest percentage of fires occurred in the Northern Hemisphere, the trend of forested NDVI increase is high. The time in which forests fully recover after fires is uncertain. Other studies have shown that early successional forests in Africa recovered within 10.2 years in the Northern Hemisphere and 12.8 years in the Southern Hemisphere [42].

A significant and complex challenge for out time is to reverse current trends in land use change due to deforestation and to recover degraded forests. By 2020, an estimated 6.2 million km^2^ of forest could be regenerated in Africa and about 113,900 km^2^ could be planted annually [87]. In this study we found that 169,899 km^2^ of forested areas showed decreasing vegetation trends and could be opportunities for restoration in areas impacted by fires. Although, our results show a recovery process in many countries or territories. Though all qualify for restoration actions for all countries, due to the results of declining trends in forest areas, many of these effort should be developed in Tunisia, Madagascar, Somalia, Morocco, and Mozambique.

Fires provide an opportunity for forest scientists, natural resource managers and policy makers to pre-define improved management actions, reconsider forest management paradigms and promote more resilient landscapes [24] amidst negative impacts of climate change and human disturbances. After fires, concerted efforts should be directed at restoring the affected landscape, including land cover and vegetation [92]. Depending on the state of degradation of an ecosystem, as well as the time of effort and financial investment, a series of restoration management approaches have been proposed, ranging from reclamation, rehabilitation, commercial reforestation/agroforestry, reforestation with native trees, assisted natural regeneration, to natural regeneration [29, 93]. However, forest landscape restoration in the African context is predominantly addressed in terms of human development [32]. Natural regeneration, the spontaneous recovery of species following disturbance, is increasingly promoted as a low-cost strategy for large-scale forest landscape restoration projects [94]. For example, in Angola, using data from field plots, evidence was found of recovery processes in the Miombo dry forests following shifting agricultural activities [95, 96]. In Tanzania, local populations restored degraded Acacia and Miombo woodlands through traditional silvopastoral practices [97]. However, some research has shown that in Africa, active revegetation practices are also effective [36]. There are numerous examples of the economic and environmental benefits of the restoration of degraded agrosilvipastoral lands for local communities by planting millions of native trees species [28].

### Relationships between seasonal spatial-temporal trend of NDVI with climatic variables in burned areas

Spatial patterns of climate and the relationship with fire have been described for the entire African continent [8], but not so for understanding the interaction between climate and post-fire vegetation recovery processes. It is well known that the climate exerts a dominant control over the spatial distribution of vegetation. Precipitation is one of the most important weather parameters across Africa, with significant variations on year-to-year to multi-decadal time-scales [19]. Previous studies have shown a corresponding response of vegetation in relation to precipitation patterns in Africa [54, 55]. Positive correlation between vegetation and precipitation has been reported throughout Africa using NDVI (1982–2015), except in deserts, and a strong negative correlation between vegetation and temperature in almost all vegetated areas except the tropical rainforest zone [98]. In addition, the correlation analyses between precipitation, temperatures and NDVI (1982–2015) in relatively dry areas in southern Africa, showed that increased precipitation supports vegetation growth, but not so in the case of temperature increase [56]. In our results we found that not all climatic variables are related to increasing or decreasing trends in NDVI values. It was observed that the lower the NDVI, the higher the maximum temperature, while the NDVI index increases in relation to higher precipitation.

In addition, we found that as vapor pressure deficit (VPD) values increased, NDVI levels decreased, with a weak linear correlation for JJA, for areas with both increasing and decreasing vegetation trends. Studies of the effects of the VPD on vegetation are very scarce. The VPD is the difference between the water vapor pressure at saturation and the actual water vapor pressure for a specific temperature [99]. Increased vapor pressure deficit is a pattern observed globally and that it is affecting the primary production of ecosystems [100, 101] because it affects plants physiologically [57] resulting in reduced vegetation growth [102, 103] and tree mortality [104].

Undoubtedly, future research could help to identify variables and factors that are relevant to identify the increase or decrease of vegetation after fire, such as fire severity, management practices, soil properties, topography, and biotic interactions. In addition, photoperiod, a factor that has been identified as dominant in the onset and end of the vegetation growing season in Africa, should be included in future analyses [105].

## Conclusions

Africa is a continent that annually concentrates thousands of fires, according to the present study, between 2001 and 2020, 8.7 million km^2^ were affected by this type of events that show marked differences between hemispheres. Considering the objectives of this study, it was established that: 1) seasonally, in the northern hemisphere fires are mainly concentrated in the months of DJF while in the Southern Hemisphere they occur in the months of JJA; 2) the highest increasing NDVI trends for both hemispheres were in the months of MAM, while for decreasing trends in JJA; 3) in the forested areas, vegetation increase trends presented the highest proportion in MAM, while decrease trends showed a higher proportion in DJF for the Northern Hemisphere and in JJA for the Southern Hemisphere; 4) of the three climatic variables, increasing vapor pressure deficit values were more related to decreasing NDVI levels. The NDVI index, which continues to be an essential tool for monitoring the state of recovery of burned areas, especially forest areas. The results presented in this study help to understanding the ecology of African ecosystems for the development of appropriate and effective actions in terms of restoration. Furthermore, it is established that the burned areas in both African hemispheres show trends of a continuous regeneration process. However, there are regions with evident degradation trends. Some countries such as Tunisia, Madagascar, Somalia, Morocco and Mozambique, deserve special attention given the magnitude of forest degradation in their respective territories. In this sense, the present study can be an input in the chain of decisions related to planning and restoration actions, as well as the generation of informed policies and management of African natural resources.

## Supporting information

S1 Table Supporting information file corresponding to Fig 3. (XLSX)

S2 Table Supporting information file corresponding to Fig 5. (XLSX)

S3 Table Supporting information file corresponding to Fig 7. (XLSX)

S4 Table Supporting information file corresponding to Fig 8. (XLSX)
